# 624. Outbreak of Human Trichinellosis Linked to Bear Meat Infected with Multiple *Trichinella* Species — Arizona, Minnesota, and South Dakota, 2022

**DOI:** 10.1093/ofid/ofad500.690

**Published:** 2023-11-27

**Authors:** Shama Cash-Goldwasser, Dustin Ortbahn, Muthu Narayan, Conor Fitzgerald, Keila Maldonado, Keila Maldonado, Keila Maldonado, Anne Straily, Sarah Sapp, Billy Watson, Yvonne Qvarnstrom, Neja Margaret, David Berman, Sarah Y Park, Kirk Smith, Stacy Holzbauer

**Affiliations:** Centers for Disease Control and Prevention, Atlanta, Georgia; South Dakota Department of Health, Pierre, South Dakota; Veterans Affairs Health Care System, Minneapolis, Minnesota; Arizona Department of Health Services, Phoenix, Arizona; Maricopa County Department of Public Health, Phoenix, Arizona; Maricopa County Department of Public Health, Phoenix, Arizona; Maricopa County Department of Public Health, Phoenix, Arizona; US Centers for Disease Control and Prevention, Atlanta, GA; Centers for Disease Control and Prevention, Atlanta, Georgia; Centers for Disease Control and Prevention, Atlanta, Georgia; Centers for Disease Control and Prevention, Atlanta, Georgia; Centers for Disease Control and Prevention, Atlanta, Georgia; Karius, St. Petersburg, Florida; Karius, St. Petersburg, Florida; Minnesota Department of Health, St Paul, Minnesota; Minnesota Department of Health, St Paul, Minnesota

## Abstract

**Background:**

In July 2022, the Minnesota Department of Health was notified of a patient with fevers, myalgias, periorbital edema, and eosinophilia; providers suspected trichinellosis. Human trichinellosis is rare in the United States and usually acquired from wild game meat. We investigated to confirm the diagnosis, ascertain additional cases, and identify the source of infection.

**Methods:**

Trichinellosis was defined as compatible symptoms in a person who consumed an epidemiologically implicated meal (probable) or had *Trichinella* antibodies (confirmed). Blood samples from exposed persons were tested for *Trichinella* antibodies and with unbiased sequencing for microbial cell-free DNA. Microscopy and a parasite detection assay based on deep sequencing were performed on suspected source meat.

**Results:**

One week before symptom onset, the patient and 8 relatives from 3 states shared a meal including meat from a bear harvested in Saskatchewan, Canada. The meat was frozen for 5 weeks until being grilled with vegetables and served rare. There were a total of 6 cases (4 probable and 2 confirmed, attack rate = 75%). Six persons had a trichinellosis-compatible illness, of whom five submitted samples for *Trichinella* antibody testing and two had positive results. Plasma samples from the two antibody-positive patients were tested for microbial cell-free DNA, and both were positive for *Trichinella* spp. DNA. Motile *Trichinella* species larvae ( >800 larvae per gram) were identified in bear meat frozen for 4 months. Deep sequencing detected 3 *Trichinella* genotypes, 2 of which matched reference sequences for *T. nativa* and *Trichinella* T6. The third genotype did not match any reference sequence; the closest match (94%) was *Trichinella* T9, which has only been detected in Japan.

**Conclusion:**

We used multimodal diagnostic testing to characterize an outbreak of human trichinellosis and confirm bear meat as the source. Freezing kills *Trichinella* species commonly implicated in pork-associated outbreaks, but *T. nativa* and *Trichinella* T6 are freeze-resistant. Consumers of wild game meat should be informed that adequate cooking is required to kill *Trichinella* parasites. Further work on identification and classification of *Trichinella* species in North America is needed.

**Disclosures:**

**David Berman, DO**, Precision Health Solutions, St. Petersburg, Florida: Advisor/Consultant

